# Characterization, Antibiofilm, and Depolymerizing Activity of Two Phages Active on Carbapenem-Resistant *Acinetobacter baumannii*

**DOI:** 10.3389/fmed.2020.00426

**Published:** 2020-08-18

**Authors:** Goran Vukotic, Mina Obradovic, Katarina Novovic, Mariagrazia Di Luca, Branko Jovcic, Djordje Fira, Horst Neve, Milan Kojic, Olivia McAuliffe

**Affiliations:** ^1^Laboratory for Molecular Microbiology, Institute of Molecular Genetics and Genetic Engineering, University of Belgrade, Belgrade, Serbia; ^2^Chair of Biochemistry and Molecular Biology, Faculty of Biology, University of Belgrade, Belgrade, Serbia; ^3^Department of Biology, University of Pisa, Pisa, Italy; ^4^Department of Microbiology and Biotechnology, Max Rubner-Institut, Kiel, Germany; ^5^Department of Food Biosciences, Teagasc Food Research Centre, Fermoy, Ireland

**Keywords:** *Acinetobacter baumannii*, carbapenem resistance, phage, biofilm, depolymerase, halo, host range

## Abstract

*Acinetobacter baumannii* is a leading cause of healthcare-associated infections worldwide. Its various intrinsic and acquired mechanisms of antibiotic resistance make the therapeutic challenge even more serious. One of the promising alternative treatments that is increasingly highlighted is phage therapy, the therapeutic use of bacteriophages to treat bacterial infections. Two phages active against nosocomial carbapenem-resistant *A. baumannii* strain 6077/12, vB_AbaM_ISTD, and vB_AbaM_NOVI, were isolated from Belgrade wastewaters, purified, and concentrated using CsCl gradient ultracentrifugation. The phages were screened against 103 clinical isolates of *A. baumannii* from a laboratory collection and characterized based on plaque and virion morphology, host range, adsorption rate, and one-step growth curve. Given that phage ISTD showed a broader host range, better adsorption rate, shorter latent period, and larger burst size, its ability to lyse planktonic and biofilm-embedded cells was tested in detail. Phage ISTD yielded a 3.5- and 2-log reduction in planktonic and biofilm-associated viable bacterial cell count, respectively, but the effect was time-dependent. Both phages produced growing turbid halos around plaques indicating the synthesis of depolymerases, enzymes capable of degrading bacterial exopolysaccharides. Halos tested positive for presence of phages in the proximity of the plaque, but not further from the plaque, which indicates that the observed halo enlargement is a consequence of enzyme diffusion through the agar, independently of the phages. This notion was also supported by the growing halos induced by phage preparations applied on pregrown bacterial lawns, indicating that depolymerizing effect was achieved also on non-dividing sensitive cells. Overall, good rates of growth, fast adsorption rate, broad host range, and high depolymerizing activity, as well as antibacterial effectiveness against planktonic and biofilm-associated bacteria, make these phages good candidates for potential application in combating *A. baumannii* infections.

## Introduction

*Acinetobacter baumannii* nosocomial infections, including bacteremia, pneumonia, meningitis, wound, and urinary tract infections, are considered as the most challenging, predominately affecting critically ill and immunocompromised patients. In recent years, outbreaks of *A. baumannii* infections, accompanied by its high resistance to antibiotics and other antibacterial agents, have resulted in an emergence of interest in this species, its virulence, transmission, environmental persistence, and treatment options (1). Of all of the ESKAPE bacterial pathogens (*Enterococcus faecium, Staphylococcus aureus, Klebsiella pneumoniae, Pseudomonas aeruginosa, Escherichia coli*), more *A. baumannii* infections are caused by multidrug-resistant strains than not (2). The rise and spread of carbapenem resistance are of particular interest since carbapenems have been the most effective antimicrobial against *A. baumannii* infections. Carbapenem-resistant *A. baumannii* infections are difficult to treat, persistent, and associated with high mortality (3). Treatment options for these cases are limited to colistin, but colistin-resistant strains of *A. baumannii* are now also emerging (4).

The World Health Organization (WHO) has listed carbapenem-resistant *A. baumannii* as a critical priority target for the research and development of new drugs (5). Bacteriophages, or viruses that infect bacteria but are harmless to eukaryotic cells, are a potential alternative to antibiotics and have received much attention in the last decade. The beginning of this decade has seen a plethora of reports on isolation and characterization of diverse *A. baumannii* phages (6–10). Apart from the phages themselves, their lytic enzymes, or endolysins, have been identified as potential biocontrol agents (11–13). *In vivo* studies showed the remarkable potential of phage application in *A. baumannii* wound infections (14, 15), pneumonia (3, 16, 17), and sepsis (18), among others. Several successful human case studies (19–21) reported on the potential of phage therapy as an approach for control of *A. baumannii* infections, as well as highlighting the need for more phage research.

The formation of biofilm by *A. baumannii* further challenges the treatment of infections because of its enhanced survival on hospital equipment, evasion of host immune system, antibiotic resistance, virulence, and relapse (2, 22, 23). One of the important traits of some *A. baumannii* phages is their ability to form translucent halos surrounding the plaques on a bacterial lawn. Halos are considered to be the macroscopic manifestations of depolymerizing activity of the phage, that is, cleavage of bacterial surface polysaccharides by phage-associated enzymes designated as depolymerases (24, 25). Given the nature of biofilms and the contribution of bacterial exopolysaccharides to their formation, it is justified to assume that depolymerases can be effective against bacterial biofilms. This was indeed demonstrated for some *Klebsiella* phages (26); however, Hernandez-Morales et al. (27) showed that biofilm of *A. baumannii* AU0783 could not be removed, even though it contained the polysaccharides targeted by phage depolymerase.

In this study, we describe two novel depolymerase producing phages, named vB_AbaM_ISTD and vB_AbaM_NOVI (from here on shortened to ISTD and NOVI, respectively), isolated from Belgrade wastewaters using carbapenem-resistant biofilm-producing clinical *A. baumannii* strain 6077/12 as a target host. Their host range was investigated against 103 clinical isolates from numerous hospitals in Serbia, for which pulsed-field gel electrophoresis pulsotypes were determined. The efficacy of phage ISTD against both planktonic and biofilm-embedded bacteria was assessed. In addition, the nature of halo formation and expansion, namely, the relation between the enlargement of halo and spreading of phages, was inspected in more detail.

## Materials and Methods

### Bacterial Strains and Growth Conditions

In total, 103 *A. baumannii* isolates from our laboratory collection were tested for evaluating bacterial susceptibility to phages (Table S1). All isolates were of clinical origin, collected in 10 different hospitals from different regions in Serbia. The majority of the isolates (*n* = 89) were carbapenem-resistant, whereas 14 were carbapenem-sensitive. Among the carbapenem-resistant isolates, three isolates were also identified as colistin-resistant.

All isolates were tested for their resistance to antibiotics using VITEK 2 automated system (BioMérieux, Marcy l'Étoile, France). Minimum inhibitory concentrations for antibiotics were determined in accordance with the guidelines of the Clinical and Laboratory Standards Institute.

All isolates were propagated either in Luria–Bertani (LB) or Mueller–Hinton (MH) broth at 37°C with aeration [180 revolutions/min (rpm)]. Stocks were made in LB supplemented with 15% (vol/vol) glycerol and kept at −80°C.

### Pulsed-Field Gel Electrophoresis

Pulsed-field gel electrophoresis (PFGE) analysis of the *A. baumannii* isolates was performed as previously described (28). *In situ* DNA was digested with *Apa*I (Thermo Fisher Scientific, 20 U/agarose block in 100 μL enzyme reaction buffer) for 3 h at 30°C. Gels (1.2% agarose) were run at LKB hexagonal electrode array PGFE system using 2015 Pulsafor unit (LKB Instruments, Bromma, Sweden) for 18 h at 300 V in 0.5 × Tris-Borate-EDTA at 9°C and stained with ethidium bromide (SERVA Electrophoresis GmbH). Pulse times were linearly increased from 8 to 18 s during electrophoresis. Gel images were captured under UV light and analyzed using Bionumerics 7.6 software (Applied Maths NV). Cluster analysis, using Pearson correlation coefficient with a 1.0% optimization and a hierarchic UPGMA algorithm, was used to generate a dendrogram describing the relationship among *A. baumannii* pulsotypes. Given the sensitivity of Dice coefficient, Pearson correlation coefficient was applied to obtain clustering without influence of minor variations in band detection. As there is no consensus on the percentage of minimum similarity needed for clustering, a minimum similarity of 60% was defined for the present data set as the cutoff value for clustering, with a minimum of two members per cluster.

### Multiplex Polymerase Chain Reactions for Identification of Sequence Type Groups

*Acinetobacter baumannii* isolates of interest were analyzed by typing using a previously described multiplex polymerase chain reaction (PCR) protocol (29).

### Phage Isolation and Plaque Assay

Phages were isolated by standard enrichment method (30) from Belgrade sewage wastewaters, using *A. baumannii* 6077/12 (31) as the host bacterium. Sewage water was centrifuged at 4,500 × *g* for 10 min at room temperature (RT) (Eppendorf 5804) and filtered through 0.45-μm filters (Filtropur S 0.45; Sarstedt, Nümbrecht, Germany). Phage enrichment was achieved by mixing 10 mL of filtrate with double strength LB in a 1:1 ratio and adding 200 μL of overnight culture of *A. baumannii* 6077/12. The mixture was incubated for 16 h at 37°C with aeration (180 rpm), followed by another round of centrifugation and filtration. Then, the filtered supernatant was collected and used for plaque assay with double-layer agar plates. Briefly, 100 μL of the filtered supernatant was mixed together with 10 μL of an overnight culture of 6077/12 in 10 mL of melted LB top agar (0.75% wt/wt agar), vortexed briefly, and poured into a Petri dish over a thin 1.5% LB agar layer.

Four rounds of single plaque purification were performed for samples giving rise to plaques. Plaques were collected using pipette tips and resuspended in SM buffer [100 mM NaCl, 8 mM MgCl_2_ × 7H_2_O, 50 mM Tris-HCl (pH 7.5)] and filtered through 0.45-μm filters. Filtrates were used for sequential plaque assay. After the final plaque assay, working phage preparations were made by inoculation of single plaque into 10 mL of mid-exponential-phase culture of strain 6077/12 followed by overnight incubation. Lysate was centrifuged and supernatant filtered as previously described.

### Phage Purification by CsCl Gradient Ultracentrifugation

A culture of *A. baumannii* strain 6077/12 was grown to mid-exponential phase in a volume of 500 mL and was infected with 1 mL of previously obtained phage suspension [titer 10^9^ plaque forming units (PFUs)/mL]. After overnight incubation, the lysate was centrifuged in Sorvall RC3B centrifuge at 13,689.2 × *g* for 30 min in rotor GS3 to pellet bacterial debris. The supernatant was collected and treated with DNaseI and RNase A (Thermo Fisher) for 2 h at 37°C. Afterward, NaCl and polyethylene glycol (PEG 8000; Sigma) were added (to obtain 1 M and 10% final concentrations, respectively), stirred, and incubated overnight at +4°C to precipitate the phages. The precipitate was centrifuged at 13,689.2 × *g* for 30 min at +4°C (Sorvall RC3B centrifuge, rotor GS3), and the pellet was resuspended in 4 mL of SM buffer. CsCl was added to reach 0.75 g/mL density. The samples were ultracentrifuged at 131,980.8 × *g* for 24 h in Beckman Coulter Optima L-80 XP ultracentrifuge in rotor SW55 Ti, after which the distinct phage-containing bluish band was visible at the midpoint of the tube. The phages were extracted from tube by using a 22-gauge needle (PrecisionGlide™; Becton Dickinson, Dubline, Ireland) and dialyzed overnight against 2 L of SM buffer at +4°C. Purified phages were filtered through 0.22-μm filters (Filtropur S 0.45; Sarstedt) and used in subsequent experiments.

### Transmission Electron Microscopy of Phage Particles

Phages purified by CsCl gradient centrifugation were dialyzed against phage buffer [20 mM Tris-HCl (pH 7.2), 10 mM NaCl, 20 mM MgSO_4_] [20-min microdialysis on 0.025 μm VSWP membrane filters (Merck Millipore, Darmstadt, Germany)]. Ultrathin carbon films (~3 × 3 mm in size) were floated from mica-sheets into a drop of phage lysate (100 μL), and after an adsorption time of 20 min, samples were transferred into a drop of 1% (vol/vol) of electron microscopy grade glutaraldehyde (20 min) for fixation. Negative staining with 2% uranyl acetate and subsequent transmission electron microscopy (TEM) (Tecnai 10; FEI Thermo Fisher Scientific, Eindhoven, the Netherlands) was done as described earlier at an acceleration voltage of 80 kV (32). Micrographs were captured with a MegaView G2 charge-coupled device camera (Emsis, Muenster, Germany).

### Phage DNA Extraction and Manipulation

DNA of both phages ISTD and NOVI was isolated either by Phage DNA Isolation Kit (Norgen Biotek) or by phenol–chloroform DNA extraction method (33). The kit was used for DNA isolation from phage lysate according to manufacturer's recommendation, whereas phenol–chloroform extraction method was performed on phages purified and concentrated by banding on the block gradient of CsCl. DNA was run on a 1% agarose gel, stained with ethidium bromide (SERVA Electrophoresis GmbH), and visualized under UV light, as previously described. Restriction enzyme digestion was performed with several enzymes: *Eco*RI, *Hin*dIII, *Apa*I, *Pst*I, *Sal*I, *Dra*I, *Xba*I, and *Dpn*I (Thermo Fisher Scientific) according to manufacturer instructions. Restriction enzyme digestions were repeated three times.

### Phage Adsorption

An adsorption test was performed to determine the number of phages that adsorb to *A. baumannii* 6077/12 host cells during a certain time period. The host strain was incubated in LB broth until exponential phase was reached. The culture was transferred into five sterile microtubes, 1 mL per tube, and 10 μL of 10^8^ PFUs/mL ISTD or NOVI phage suspension was added. Mixed suspensions were incubated for 1, 2.5, 10, 20, and 30 min at 37°C without aeration, to allow adsorption of phage particles to the bacterial cell surface. Suspensions were then centrifuged for 1 min at the speed of 15,000 × *g* (Eppendorf 5418 R), so the bacterial cells with phages attached would be harvested in a pellet. The supernatant was transferred into sterile microtubes and was used in a plaque assay for determination of the unadsorbed phage titer. Based on the starting concentration of phages added and the concentration of unadsorbed phages, the percentage of adsorbed phages was calculated by the formula of Geller et al. (34):

$$\begin{array}{l} \text{Adsorbed phages}\left(\%\right)\\ \;=\left(1-\text{titer of supernatant}/\text{stock titer}\right)\times 100. \end{array}$$

### One-Step Growth and Burst Size

The life cycle of phages ISTD and NOVI was investigated by a one-step growth experiment, and “burst size” was also determined as the number of virions released from one cell. *Acinetobacter baumannii* 6077/12 was inoculated in 10 mL of LB broth and incubated at 37°C, with aeration (180 rpm), until it reached exponential phase. Culture volume of 1 mL was transferred to a sterile microtube and was infected with 10 μL of ISTD or NOVI phage suspension [stock concentration 10^8^ PFUs/mL, multiplicity of infection (MOI) 0.01]. The mix was incubated for 5 min at 37°C, to allow the adsorption of phages to the bacterial cell surface, and afterward diluted 100 × in LB broth, in order to avoid contact of the unadsorbed or newly liberated phages with new cells. During further 60-min incubation, starting from the moment of dilution, a 100-μL sample of the suspension was collected every 10 min, and phage titer was determined. Burst size was calculated as the ratio between the number of total released phages and the number of infected bacterial cells.

### Host Range

Host range of ISTD and NOVI was investigated by testing their lytic activity on 103 *A. baumannii* isolates in total. Sensitivity toward the phage was tested by double agar layer spot assay. Briefly, 10 μL of overnight culture of each isolate was inoculated into 10 mL of melted soft agar. After vortexing, soft agar containing bacteria was overlaid on LB agar in Petri dish. Upon solidifying, 10 μL of phage suspension was spotted on top of the agar. After 16 h of incubation at 37°C, plates were inspected for appearance of growth inhibition zones surrounded by halos. Isolates that were sensitive to phages were additionally inspected after 24, 48, and 72 h at RT for enlargement of halos.

### Halo Dynamics

The relation between the spreading of halo and spreading of phages through the agar was investigated using similar approach as described in the work of Cornelissen et al. (35). The abundance of phages, as well as the bacteria, was evaluated inside the plaque, in the plaque vicinity, and in more distant parts of halo formed with a regular spot assay. The values were addressed as PFUs and colony-forming units (CFUs) per gram of agar. To extract the cells and phages, parts of soft agar were sliced with a sterile scalpel, measured for weight, and resuspended in 1 mL of saline solution by vigorous vortexing. From this mixture, serial dilutions were made and plated for CFU counting. For PFU counts, a drop of chloroform was first added to the solution in order to kill the residual bacteria, and then the serial dilutions were made. Obtained results were pondered according to the weight of the agar pieces. Statistical analysis of bacterial and phage titers was done by one-way analysis of variance followed by Tukey *post-hoc* test (Statistica Software).

Additionally, the ability of phages to form halos on pregrown bacteria was tested. Bacteria were added to melted soft agar, poured, and grown overnight. The following day, 10 μL of phage preparation was dropped on the surface of the lawn; the plate was incubated at 37°C for additional 24 h, after which the plates were inspected for zone or halo formation.

### Bactericidal Kinetics of ISTD

Characterization of phage-planktonic bacteria kinetics was performed as follows: overnight culture of bacterial strain *A. baumannii* 6077/12, grown in MH, was adjusted to density of 0.5 McFarland scale and further on diluted 100 times into 200 μL MH broth without (growth control) or with ISTD phage suspension in a 96-well-microtiter plate (Tissue Culture Plate; Sarstedt). Phages were applied at MOIs 0.01, 0.1, 1, and 10. At 3-, 6-, 9-, and 24-h time points after treatment, samples were taken, serially diluted in saline, and plated on MH agar. Viable cell counts were calculated from triplicate assays.

### Biofilm Formation and Antibiofilm Activity of ISTD

Biofilm formation assay was performed according to the method described previously (36). Briefly, overnight cultures of *A. baumannii* isolates were adjusted to density of 0.5 McFarland scale, and 20 μL was transferred to wells of 96-well-microtiter plates filled with 180 μL LB broth medium. Microtiter plates were incubated aerobically for 48 h at 37°C. After incubation, attached bacteria were washed three times with phosphate-buffered saline (PBS; pH 7.2) and fixed by drying 30 min at 65°C. For staining and visualization of biofilm, 0.1% crystal violet (HiMedia Labs Pvt. Ltd., India) was used (30 min, RT). The stain was rinsed by washing three times with PBS and then resolubilized with mixture of ethanol (96%) and acetone (4:1). Quantification of biofilm formation was done by measuring absorbance at 595 nm using Plate Reader Infinite 200 pro (MTX Lab Systems, Austria) from decuplicate assays.

Antibiofilm activity of phages was evaluated against biofilm formed on porous glass beads having a diameter 4 mm, pore size 60 μm, and surface area of ~60 cm^2^ (VitraPor; ROBU, Hattert, Germany) as described previously (37, 38). Briefly, overnight culture of *A. baumannii* 6077/12 was diluted 100 times in MH and incubated with glass beads (1 bead: 1 mL) for 24 h, at 37°C without aeration. Then, beads were washed three times with saline solution, and each bead was transferred in a separate well of a 24-well-plate (Tissue Culture Plate; Sarstedt) for treatment. Each treatment was done in triplicate. Beads were incubated with phages at two MOIs–0.1 and 100—in MH broth at 37°C. An untreated control of biofilm without phages was also prepared. After 6- and 24-h treatments, CFUs of biofilm-embedded bacteria were counted. Each bead was washed three times with saline and transferred into tubes with 1 mL of sterile saline. Tubes with beads were vortexed for 30 s, sonicated in an ultrasound water bath for 60 s, and again vortexed for further 30 s to dislodge bacterial cells from bead surface, as previously described (39). Further on, serial dilutions were made and spotted on MH agar plates. Cells were counted from biofilm before treatment, biofilm after phage treatments, and biofilm with fresh medium. After 24 h of incubation, the plates were inspected, and numbers of colonies were counted.

## Results

### Phage Isolation, Plaque, and Virion Morphology

**Figure 1:**
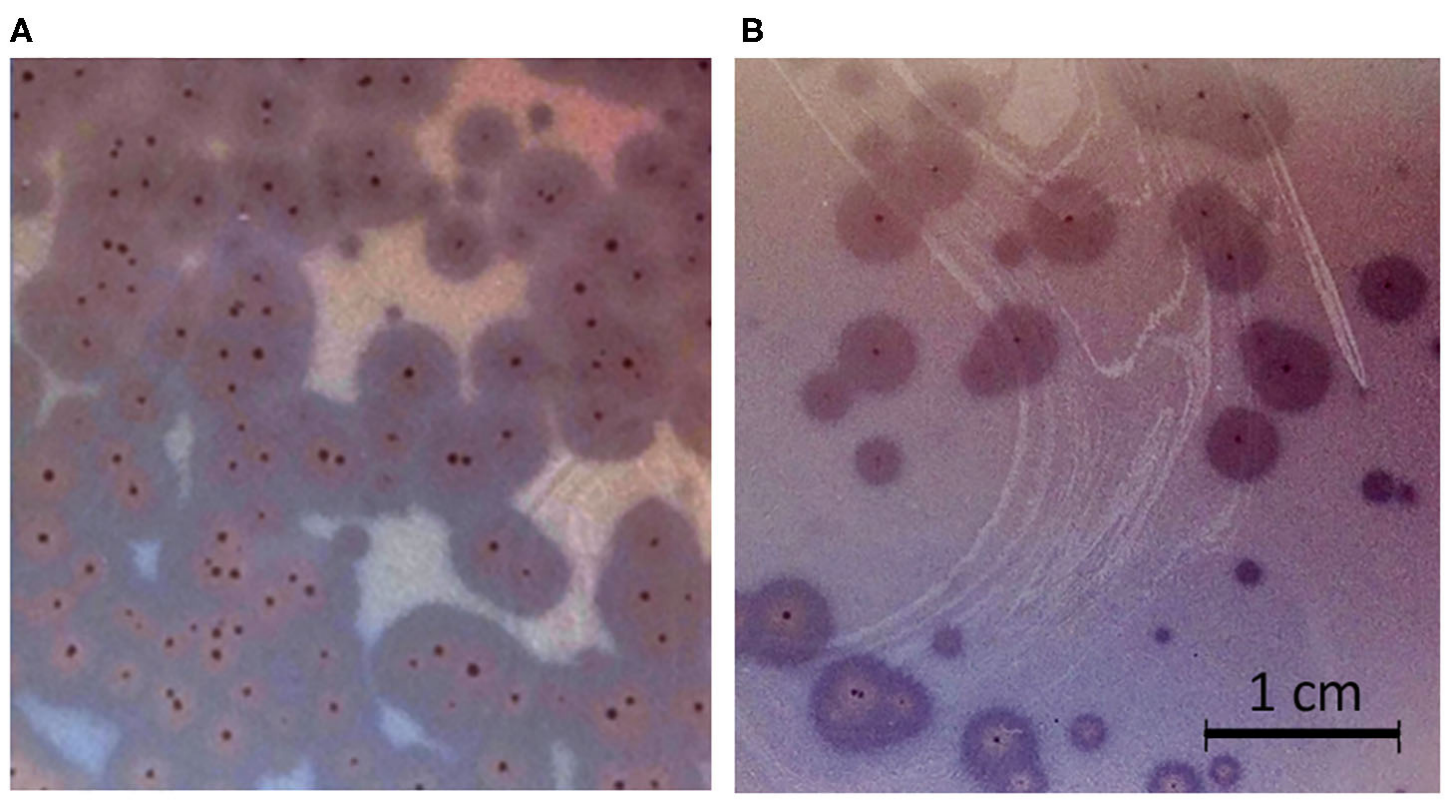
Plaque morphology. **(A)** Phage ISTD. **(B)** Phage NOVI.

Phages ISTD and NOVI were isolated from two sewage wastewater samples, using *A. baumannii* 6077/12 as the host bacterial strain, given its overall characteristics (31) and good biofilm-forming ability (Figure S2). Using phage purification methods, concentration, and titration, a pure, high-titer (10^11^ PFUs/mL) stock of both *A. baumannii* phages was obtained. Plaques produced by both phages were small, <1 mm in diameter, surrounded by large (5–6 mm) halos (Figure 1), which were constantly enlarging 2–3 mm/d.

**Figure 2:**
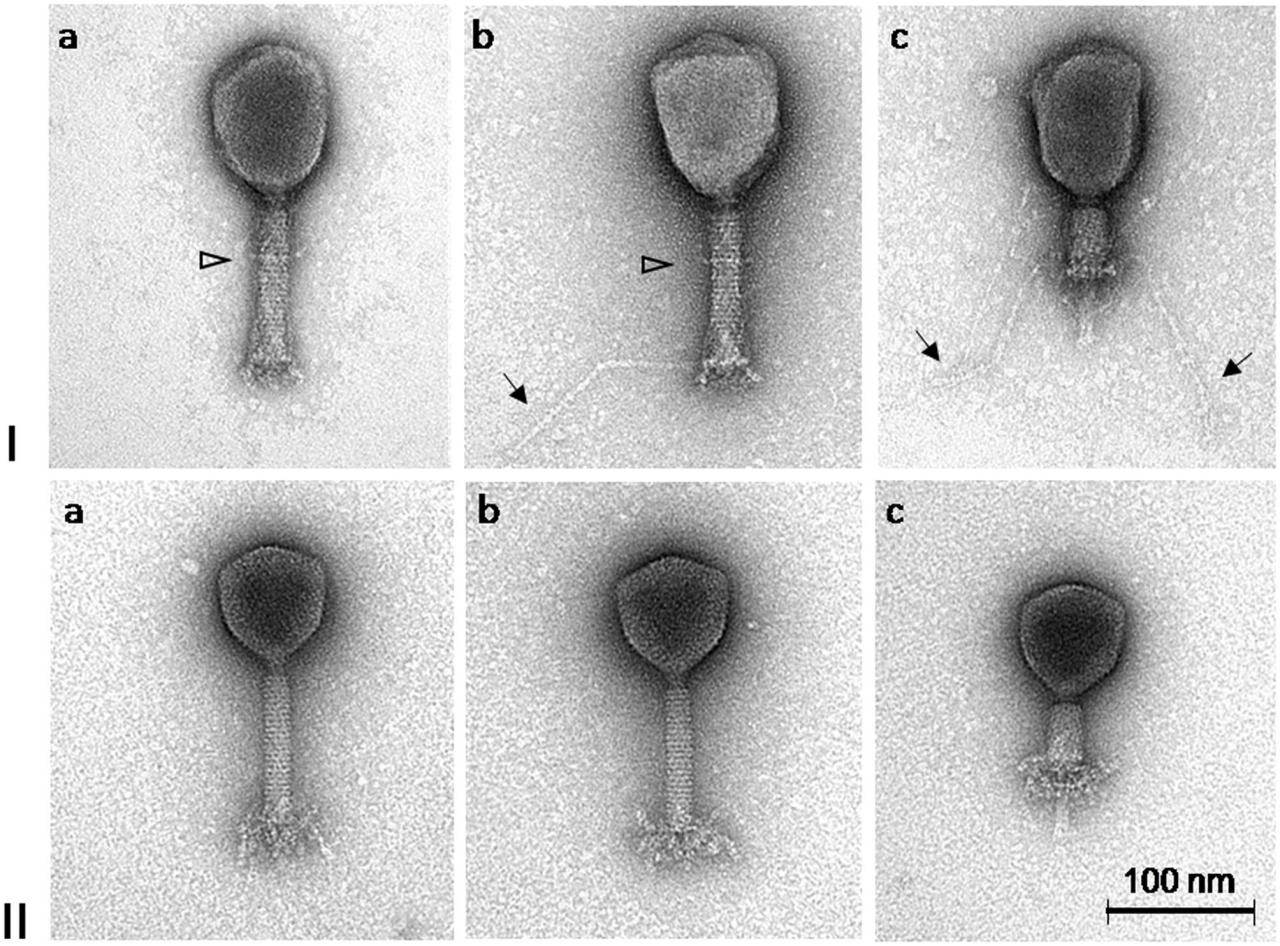
Transmission electron micrographs of *A. baumannii* phages ISTD **(I)** and NOVI **(II)** negatively stained with 1% (wt/vol) uranyl acetate. Extended long tail fibers of phage ISTD are indicated by arrows in Ib and Ic. A distinct ring structure on the tails of phage ISTD is indicated by a triangle (Ia and Ib). Phages with contracted tail sheaths and protruding tail tubes are shown in Ic and IIc, respectively. Head dimensions: ISTD, 113.9 ± 4.4 × 83.0 ± 4.0 (*n* = 10); NOVI, 83.0 ± 1.8 (*n* = 8). Tail length incl. bp appendages: ISTD, 123.3 ± 1.8 (*n* = 9); NOVI, 125.7 ± 2.9 (*n* = 8). Tail length without bp appendages: ISTD, 110.0 ± 1.8 (*n* = 10); NOVI, 107.2 ± 2.2 (*n* = 8). Tail width: ISTD, 21.8 ± 1.3 (*n* = 9); NOVI, 19.6 ± 0.5 (*n* = 8). Long tail fiber length: ISTD, 148.7 ± 10.6 (*n* = 17); NOVI, N/A. All dimensions are given in nm.

Based on the TEM micrographs, both phages have rigid, thick, contractile tails of similar size but different head morphotypes, that is, phage ISTD with prolate heads, and phage NOVI with isometric heads (Figure 2). Their morphology suggests that both phages belong to the order Caudovirales, family Myoviridae. For phage ISTD, long tail fibers are typically folded upward along the tail sheath (Figure 2Ia) but are occasionally also visible in random extended positions with typical knee-like bends (Figures 2Ib,c). A distinct ring structure is also visible on the tail (Figures 2Ia,b). For phage NOVI, short (flexible) tail appendages are visible at the bottom of the phage tail (Figures 2IIa–c). Both phages are also shown with contracted tail sheaths and protruding tail tubes in Figures 2Ic, IIc, respectively. The relevant phage dimensions are summarized in the legend of Figure 2.

### Isolates Genotyping and Host Range Analysis

All tested *A. baumannii* isolates and their characteristics are given in Table S1. Pulsed-field gel electrophoresis analysis was performed for all 103 *A. baumannii* isolates. Using the *Apa*I macrorestriction patterns generated, cluster analysis was performed using Bionumerics software, and the results demonstrated high heterogeneity among the analyzed isolates. Thirteen groups designated A–M could be identified, along with 15 strains producing unique pulsotypes, which could not be clustered within the mentioned groups (Figure S1). Groups B and F were predominant, comprising 22 and 20 isolates with differences up to 37.8 and 39.6%, respectively. Other groups consisted of relatively few isolates (A: 3, C: 5, D: 8, E: 2, G: 3, H: 4, I: 2, J: 7, K: 2, L: 7, M: 3).

Phage ISTD lysed 37 isolates (~36%), whereas NOVI lysed 23 isolates (~22%). Interestingly, phage-sensitive and phage-resistant isolates could be found in almost all groups, suggesting that sensitivity to these phages is not associated with the genetic relatedness of the isolates. However, it should be noted that groups G, J, and K (12 isolates in total) included only phage-sensitive isolates, whereas groups H, L, and M (14 isolates in total) consisted only of phage-resistant isolates.

In addition, 37 ISTD-sensitive isolates were tested using multiplex PCR to establish their sequence type groups. Typing revealed that 29 of these isolates belonged to sequence type Group 1, which corresponded to European clonal complex II, whereas eight belonged to sequence type Group 3, which corresponded to European clonal complex III (Table S1). In total, 23 isolates were sensitive to both phages, mostly belonging to ECC II.

Moreover, it was demonstrated that isolates with the same PFGE profile have different sensitivity toward the phage ISTD. For instance, pairs 8709–11462, 1865/12–8778, GN228–GN385 have the same pulsotype (*Apa*I macrorestriction profile), but exhibit different phenotype regarding phage ISTD resistance/sensitivity.

All sensitive isolates produced lysis zones of the same size and clearness. Halos appeared around all plaques; however, the timing of the appearance of the halo differed. With several isolates (16F, 12655, 13056, 8761, 10061, 6000, 8709, 9977, 11587, GN227), the halo from both phages did not appear after the first 24 h of incubation, but did so after prolonged incubation. These isolates have not been grouped together according to PFGE or sequence type. Among carbapenem-resistant isolates, two colistin-resistant isolates (GN1106 and GN1107) were also sensitive to both NOVI and ISTD phages.

### Phage DNA Analysis

In order to determine the genetic basis of the phenotypic differences of these two phages, total DNA was isolated and analyzed. DNA of both phages was successfully isolated, and its integrity was confirmed with agarose gel electrophoresis (data not shown). However, none of the eight tested restriction enzymes used were able to cut either DNA. Also, four attempts to sequence the genomes using different platforms of next-generation sequencing (Illumina HiSeq/MiSeq and PacBio's RS II) failed to generate any sequence data.

### Phage Adsorption and One-Step Growth Curve

**Figure 3:**
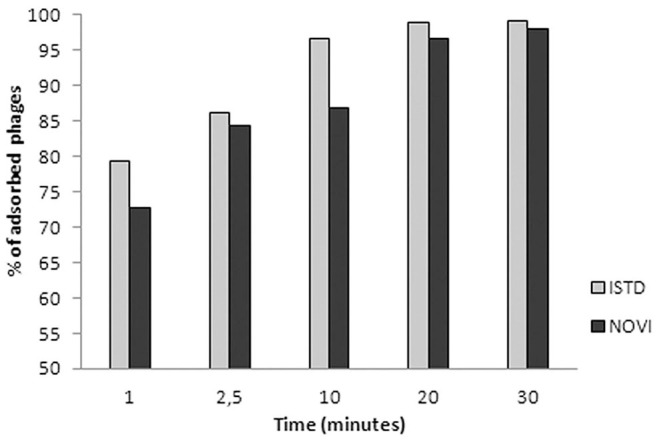
Adsorption rates of phages ISTD and NOVI on *A. baumannii* 6077/12. Bars indicate the percentage of adsorbed phages at different time points. Each bar represents the mean of three independent measurements.

In Figure 3, the adsorption rates of both phages are reported. After 1 min of phage–bacteria incubation, 79 and 73% of ISTD and NOVI phage particles were attached to host cells, respectively, and after 30 min, more than 95% of particles of both phages were adsorbed.

**Figure 4:**
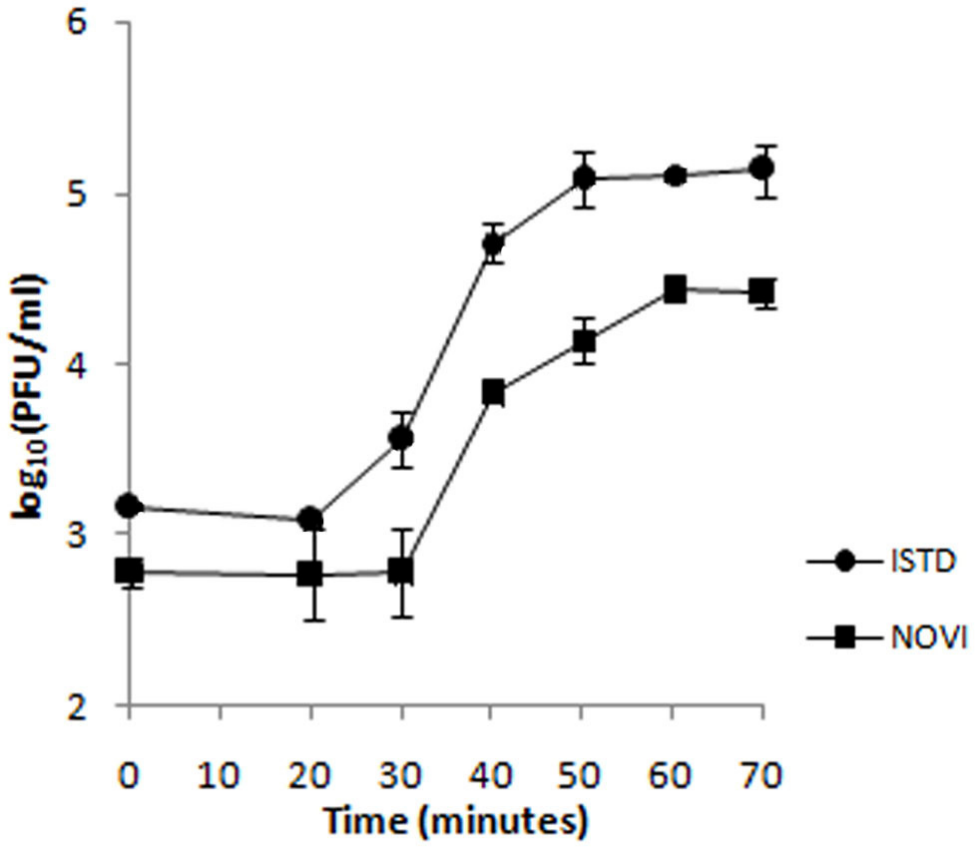
One-step growth curve of phages ISTD and NOVI on *A. baumannii* 6077/12. Phages were grown in an exponential phase culture of *A. baumannii* 6077/12. Data points indicate the PFUs/mL at different time points. Each data point represents the mean of three independent measurements.

To identify the different phases of the phage infection process, a one-step growth curve of both phages on *A. baumannii* 6077/12 was determined (Figure 4). The latent period (the period of time that passes from the initial infection to the first burst of new phages) is 20 min for ISTD and 30 min for NOVI. After an exponential rise of PFUs/mL, ISTD reaches a plateau after 50 min and NOVI after 60 min. ISTD has a burst size of 113 ± 7, and NOVI of 52 ± 6 phage particles released per bacterial cell.

### Halo Dynamics

As mentioned, both phages produced turbid halos around the plaques on the majority of tested sensitive isolates, indicating depolymerizing activity of phage enzymes on surface exopolysaccharides of the given isolate. It was noted that exopolysaccharides were efficiently degraded, considering that halo increment could be monitored visually. To establish whether free phages or diffusing enzymes are responsible for this phenomenon, the halo was investigated for phage presence and titer. In parallel, the number of viable bacteria was also monitored.

**Figure 5:**
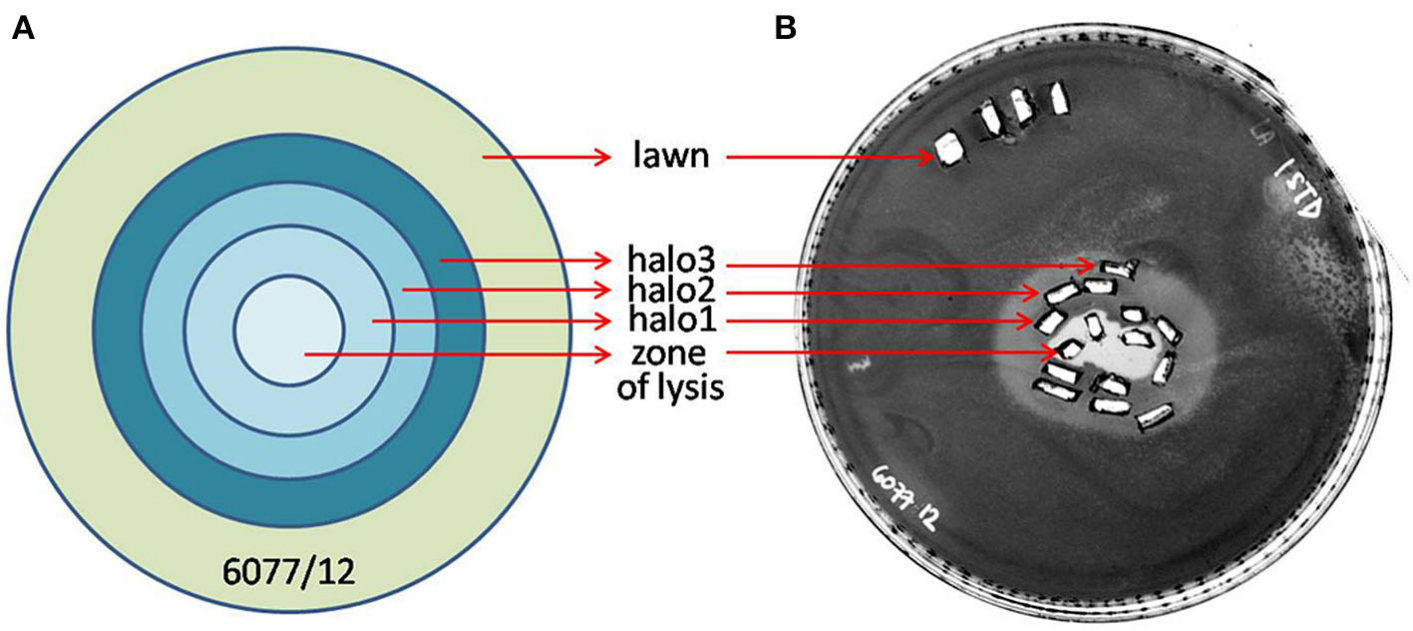
Graphic depiction of halo enlargement **(A)** and the Petri dish that was analyzed **(B)**. Samples were taken in duplicate each day for 3 consecutive days, and PFUs and CFUs were counted and pondered according to the weight of the agar pieces. As controls, the unaffected lawn area and zone of lysis were analyzed in the same way.

**Figure 6:**
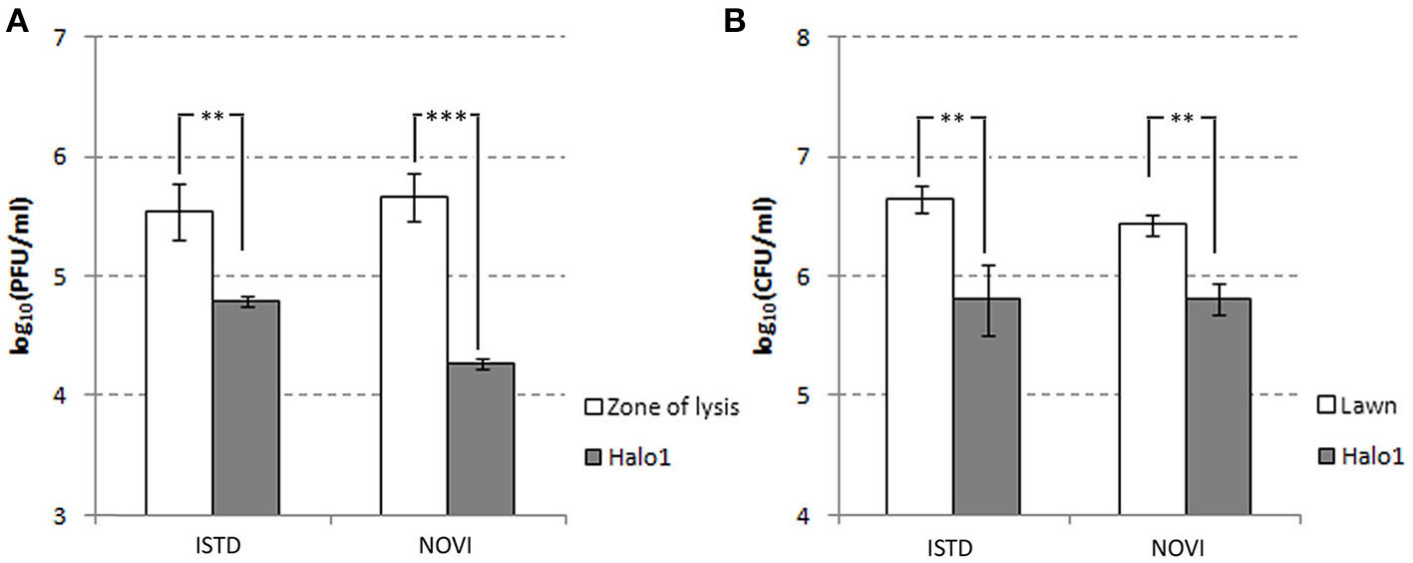
Halo dynamics. Change in phage and cell count was monitored in halo1 (see Figure 5), the zone of halo proximal to the zone of lysis. The one-way analysis of variance with Tukey *post-hoc* test was used to compare the phage titer **(A)** and cell count **(B)** data. Significant differences are indicated by asterisks (**p ≤ 0.01; ***p < 0.001). Data are presented as the mean ± standard deviation.

The halo around the zone of lysis produced by a regular spot assay expanded over the incubation period, with the diameter of the halo increasing by 2–3 mm each day. These “new” layers of halo, named halo1, halo2, and halo3 (Figure 5), were analyzed over three consecutive days, as the halo was enlarging. Free phages could only be detected in halo1, that is, in proximity of the spot, whereas halo2 and halo3 did not contain free phages. The number of phages found in halo1 was relatively high, as, for example the titer of ISTD amounted 6.3 × 10^4^ PFUs/mL compared to 3.5 × 10^5^ PFUs/mL in the zone of lysis. To determine if this phage presence in halo1 interfered with the present bacteria, bacterial number was also monitored. Viable cell count in halo1 was 6.3 × 10^5^ CFUs/mL compared to 4.4 × 10^6^ CFUs/mL in the unaffected bacterial lawn (Figure 6). The titers of both phages and bacteria remained unchanged during the next 2 days, except for the slight decrease in CFUs, which was also noted in control (data not shown). Comparing the performance of two phages inside halo1, it seems that ISTD diffuses more vigorously and results in higher reduction of bacterial cell number than phage NOVI.

**Figure 7:**
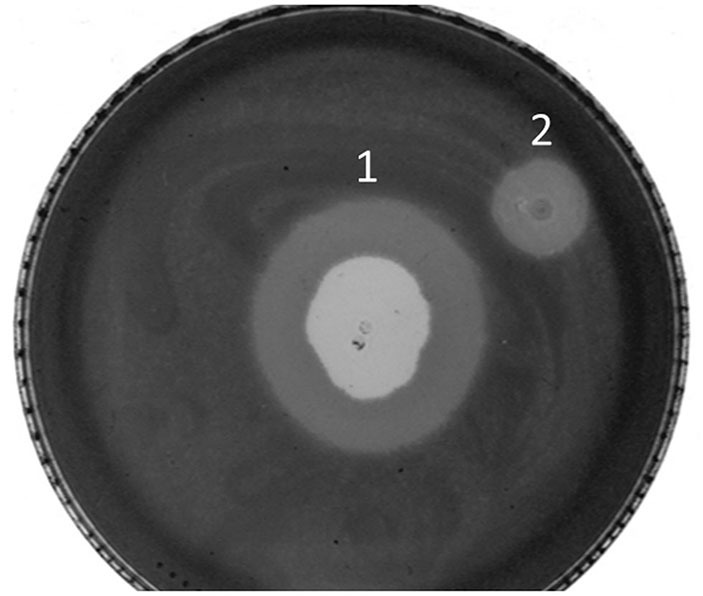
Plaque and halo formation on *A. baumannii* 6077/12. (1) Zone of lysis and halo after spotting ISTD phages on bacteria in exponential growth phase, (2) halo after spotting ISTD phages on bacteria in stationary growth phase.

Following the results obtained in the halo analysis, the possibility of lysis zone or halo forming was analyzed on pregrown bacterial cells, on stationary phase of growth. The experiment was set as usual double-layer spot test, and phages were spotted on top of the soft agar immediately after it solidified but also on the following day, after the bacterial lawn was already formed by growth overnight at 37°C. The phages spotted immediately after solidifying of agar produced the usual zone of lysis and the halo, which spread with time, but phages spotted on a preformed, 24-h bacterial lawn were also able to produce the spreading halo, without a visible zone of lysis (Figure 7). This result was confirmed by additional experiments, where it was shown that phages, even when applied 4 days after the formation of the bacterial lawn, do not produce zones of lysis but can produce a halo, which spreads with time (data not shown).

### Bactericidal Kinetics

**Figure 8:**
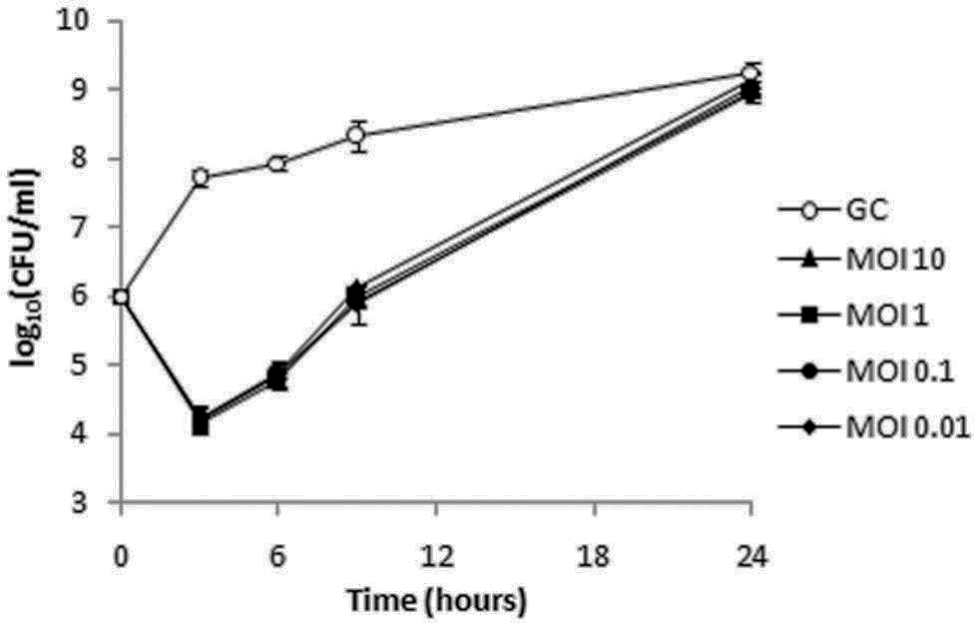
Bacterial cell counts after treatment of planktonic culture of *A. baumannii* 6077/12 with bacteriophage ISTD. A reduction of 3.5 log units in CFU number compared to the untreated control was observed 3 h after treatment. After 24 h, no difference in the CFU counts between the infected cultures and growth control was observed. All MOIs applied had the same effect. GC, growth control without phages.

Given the shorter latent period, higher burst size, and broader host range, ISTD phage was selected for further analysis of its killing efficiency on bacterial cells during either planktonic or biofilm lifestyle. The activity of ISTD phage against planktonic *A. baumannii* 6077/12 cell culture was evaluated. *Acinetobacter baumannii* 6077/12 cells in early exponential phase of growth were incubated with different titers of phage, at MOIs of 0.01, 0.1, 1, and 10, respectively. Interestingly, all MOIs applied yielded the same results regarding the host lysis kinetics (Figure 8). After 3 h, the number of planktonic cells was almost 100-fold lower in comparison to the inoculum size of cells, and a reduction of 3.5 log units in CFU number was observed when the phage-treated samples were compared with the untreated controls. By 6 h, the cell count started recovering, but the reduction was still 3 log units compared to untreated growth control. However, after 9 h, the difference between treated and untreated bacteria decreased to 2.3 log units, whereas after 24 h, there was no difference in the CFU count between the infected cultures and growth control.

### Antibiofilm Effect

**Figure 9:**
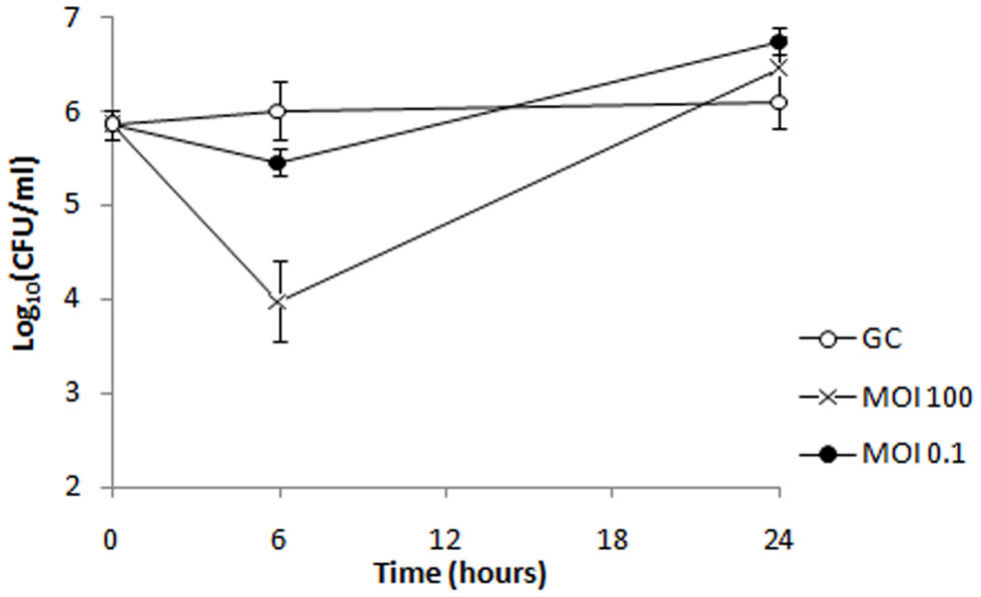
Bacterial cell counts after treatment of biofilm formed by strain 6077/12 with bacteriophage ISTD. A reduction of viable cell count of 0.6 and 2 log units was observed 6 h after treatment of biofilm with phage ISTD at MOI 0.1 and MOI 100, respectively, whereas after 24 h, no difference in CFU numbers was observed between treated samples and untreated control. GC, growth control without phages.

Phage ISTD bactericidal activity was also evaluated against 6077/12 cells embedded in biofilm formed on porous glass beads. Phages at MOIs 0.1 and 100 were incubated with bead biofilms, and the CFUs representing cells from biofilm were counted at 6 and 24 h posttreatment. After 6 h of bacteria incubation with ISTD phages at MOIs 0.1 and 100, respectively, the number of viable cells dislodged from biofilms was 0.6 and 2 log units lower in comparison to the untreated control, respectively (Figure 9). However, after 24 h, no difference in CFUs number was observed between treated and untreated controls.

## Discussion

During the last decade, there has been an alarming global increase in the incidence and prevalence of carbapenem-resistant Gram-negative bacteria. One of the most threatening is *A. baumannii*, which causes severe infections associated with a high rate of morbidity and mortality. Moreover, it is a bacterium prone to easily acquire resistance to the different classes of antibiotics, resulting in difficult-to-treat infections. In 2017, the WHO listed carbapenem-resistant *A. baumannii* as a critical target for which the research and development of new antibiotics are a priority (5). The lack of currently effective antibiotics or antibiotics in development has prompted the US Centers for Disease Control to escalate its threat level from “serious” to “urgent” in 2019 (40). The situation demonstrates the clear need for alternative solutions.

Recently, bacteriophages, as natural enemies of bacteria, reemerged as one of the potential solutions for controlling bacterial infection. They present promising agents to be applied in the therapy of infectious diseases, even as agents of last resort for treating diseases caused by multidrug-resistant pathogens, as evidenced by a growing body of research data and publications on both natural and engineered phages (21, 41, 42). However, there is still a strong need to further deepen our fundamental understanding of phage biology and research on their potential application.

The phages characterized in this study, ISTD and NOVI, were isolated using clinical carbapenem-resistant, biofilm-producing *A. baumannii* 6077/12 host strain. Both phages belong to order Caudovirales, family Myoviridae, and possess strong depolymerase activity. Until recent work on vB_AbaM_B9, (43), this has been reported only in members of Podoviridae family. Both phages adsorb efficiently, have a short latent period, and cause complete lysis of host strain 6077/12. Host range assessment showed that both phages can infect various unrelated strains. Given that one of the most important obstacles for phage therapy is the natural narrow specificity of phage host range, we analyzed the susceptibility of more than 100 clinical *A. baumannii* isolates to assess the host range of the two phages. The isolates were collected over several years in hospitals from different geographic areas in Serbia and displayed diversity belonging to 13 clusters and 15 unique single pulsotypes. No association between the PFGE patterns, clonal complex typing or antibiotic resistance profiles, and sensitivity to phages could be deduced. The most intriguing result was the difference in phage sensitivity displayed by the same pulsotypes (isolates 8709-11462, 1865/12-8778, and GN228-GN385). These three pairs of isolates, sharing the same PFGE profile, showed different sensitivity toward the phage attack. This implies that differences between these isolates are very restricted, but may include phage resistance mechanisms. This also indicates that phage sensitivity is an isolate-specific characteristic and that this should be considered when determining a phage's host range.

As already demonstrated in several studies, host range of phage depolymerases is the same as the host range of phages themselves (24, 44). This is the reason why it has been postulated that depolymerases are responsible for host recognition and attachment, which was proven through phage engineering (45). Our results are in line with these findings; however, we noted that phages did not produce halos on all sensitive strains with the same efficiency. The majority of sensitive strains produced 2- 3-mm-diameter halos after the first 16 h of incubation, with an additional 2 to 3 mm being enlarged every 24 h. Ten isolates (~28%) produced halos with much slower rate, which might imply they produce somewhat different exopolysaccharides that are still cleaved by phage depolymerases, but perhaps in a slower manner.

There are two hypotheses explaining the halo spread, which is a characteristic of depolymerase-containing phages (46). One is that it is a consequence of diffusing offspring virions, and the other is that soluble enzymes are mediating the phenomenon. Based on the results from our study, it is clear that the independent enzyme molecules are diffusing through the agar even without phage multiplication, with several important findings. First, it is important to note that phages could be detected outside the plaque, even in high numbers, which suggests they are indeed capable of diffusing through the agar. However, they are concentrated only in the narrow region nearest to the plaque and cannot be detected in further regions, albeit the halo spreads and eventually cover the Petri dish. Second, it is important to note that the bacterial load in the same region dropped for almost one order of magnitude, which is a possible indication of infection and lysis.

Sequencing of phage genomes presented a tremendous obstacle in our work. Although four different sequencing companies made efforts using different approaches for library construction, including Nextera and PacBio RS II technologies, no reliable results were obtained, mainly because of failure at representative library construction steps. This, coupled with resistance of phage DNA to restriction enzymes digestion, points to high level of DNA modifications. Phage DNA modification is not an unknown phenomenon, with the most commonly described being the adenine and cytosine methylation (47, 48). However, it should be noted that ISTD and NOVI DNAs were not digested with the *Dpn*I restriction enzyme that cleaves only when its recognition site, GATC, is methylated. A similar problem with restriction enzyme digestion was reported by Kusradze et al. (14) in their work on *A. baumannii* phage vB-GEC_Ab-M-G7, which encodes a 90-kb genome. This problem might be overcome by amplifying the genome using enzyme-free denaturation steps, and this work is in progress.

Both phages possess similar morphology, belong to the same family, have a similar replication cycle, and are active on a similar portion of isolates analyzed. In total, 23 isolates were sensitive to both phages.

However, ISTD performed better regarding some of the analyzed characteristics. It displayed better adsorption rate to bacterial cells than NOVI, especially in the first minute of adsorption. It also demonstrated a more effective lytic cycle producing more progeny in a shorter time interval. In addition, its host range encompassed the host range of phage NOVI but also additional isolates. Having in mind all mentioned, we decided to analyze phage ISTD lytic cycle in more detail, as well as its ability to lyse biofilm-embedded cells.

The bactericidal efficacy of phage ISTD against planktonic bacteria, or a time-kill study, demonstrated significant reduction in cell number followed by exponential regrowth, a phenomenon described in literature for several *A. baumannii* phages. For example, in similar experiments, Regeimbal et al. (49) noticed regrowth in the fourth hour postinfection, whereas Liu et al. (45) and Peng et al. (50) observed it in the eighth hour. The explanation of observed regrowth in all mentioned studies came from the observation that the bacterial cells that appeared after 24 h were resistant to phage attack.

A similar phenomenon was observed in the antibiofilm assay. The starting cell number to which phages were applied was about the same as in the assay with planktonic bacteria; however, the CFU decrease after phage application was not as prominent as with planktonic cells. This is understandable, first because the biofilm by definition protects cells from any agent that might affect them. Also, and very importantly, forming of biofilm took 24 h prior to the treatment, which indicates that bacteria progressed from exponential to stationary phase of growth, that is, lower level of metabolic activity. This was corroborated with a very slight increase in cell number in control group during 24 h of experiment trial. Nevertheless, the reduction observed in the sixth hour after treatment suggests that phages are active on biofilm-embedded cells.

Interestingly, influence of phage MOI on cell survival varied between the two experiments. Although we did not test higher MOIs, the identical kinetics, and overall bactericidal effect of four different MOIs applied against planktonic cells suggest that the effect is not dose-dependent. On the other hand, 2 MOIs applied against biofilm produced results that differed significantly. As mentioned, several studies demonstrated that relatively fast planktonic regrowth originated from phage-resistant bacterial cells. This phenomenon offers plausible explanation of our results, considering that even the smallest applied MOI provided enough phages to lyse all susceptible bacteria in plankton. A higher MOI could not produce better results, as the resistant bacteria survive irrelevantly of number of applied phages. However, with biofilm bacteria, the situation is different, as bacteria are much less susceptible to phage infection for reasons already explained. More phages in this setup mean higher probability of metabolically active bacterial cell binding the phage, but also of phenomenon called “lysis from without,” that is, cell destruction that is mediated exclusively by virion adsorption (51). Virion adsorption achieved with a greater MOI, without the replication of virus inside the cells, can lead to cell death regardless of its metabolic inactivity characteristic of biofilm bacteria.

Taking the results from both experiments, it could be concluded that phage application leads to severe reduction in bacterial viable cell count. Nevertheless, the bacterial cells surviving the attack are able to restore normal growth in the matter of hours. Although it might appear that no net effect was achieved with phage treatment, as bacteria recovered fully and reached their full growth capacity, superimposed with phage resistance, these results mean that phage treatment provides a window in which bacteria are rendered to the cell count eradicable by some other antimicrobial agent, presumably another phage, antibiotic, or some other antimicrobial agent, and this will be the object of our further research.

In conclusion, both phages isolated in this study demonstrated efficiency at lysing various *A. baumannii* strains, moderately wide host range, high adsorption rate, short latent period, and high burst size; however, phage ISTD performed better with respect to all the mentioned characteristics. It was active on carbapenem- and colistin-resistant clinical stains of *A. baumannii*, and it yielded several log cell reduction in both planktonic and biofilm associated bacteria, which makes it a good candidate for phage therapy by designing phage cocktails for application. This study represents an important step in the discovery and analysis of new phages that may contribute to the more successful therapy of diseases caused by the multidrug-resistant pathogen *A. baumannii*. Given the difficulty of genome sequencing, which requires an interdisciplinary approach, and the specificities of each pathogen and their phages, there is a growing need for global collaboration between basic scientists, services, and clinicians to overcome any stumbling blocks in the use of phages in therapy.

## Data Availability Statement

The raw data supporting the conclusions of this article will be made available by the authors, without undue reservation.

## Author Contributions

GV, MO, KN, and MD contributed to acquisition and analysis of data. GV, BJ, MK, DF, HN, and OM contributed to design of the work, analysis, and interpretation of the data. GV, MO, MD, and OM drafted the manuscript. MK contributed to the conception of the work and critically revised the final version to be published. All authors contributed to the article and approved the submitted version.

## Conflict of Interest

The authors declare that the research was conducted in the absence of any commercial or financial relationships that could be construed as a potential conflict of interest.
