# Supplementary material for: Characterization, Antibiofilm, and Depolymerizing Activity of Two Phages Active on Carbapenem-Resistant Acinetobacter baumannii
Source: Front Med (Lausanne). 2020 Aug 18;7:426. doi: 10.3389/fmed.2020.00426 (PMC7461965; doi:10.3389/fmed.2020.00426)
Supplement: Supplementary file 1 [file Data_Sheet_1.docx]

Supplementary Material

# 1. Supplementary Figures and Tables

**1.1 Supplementary Figures**

**
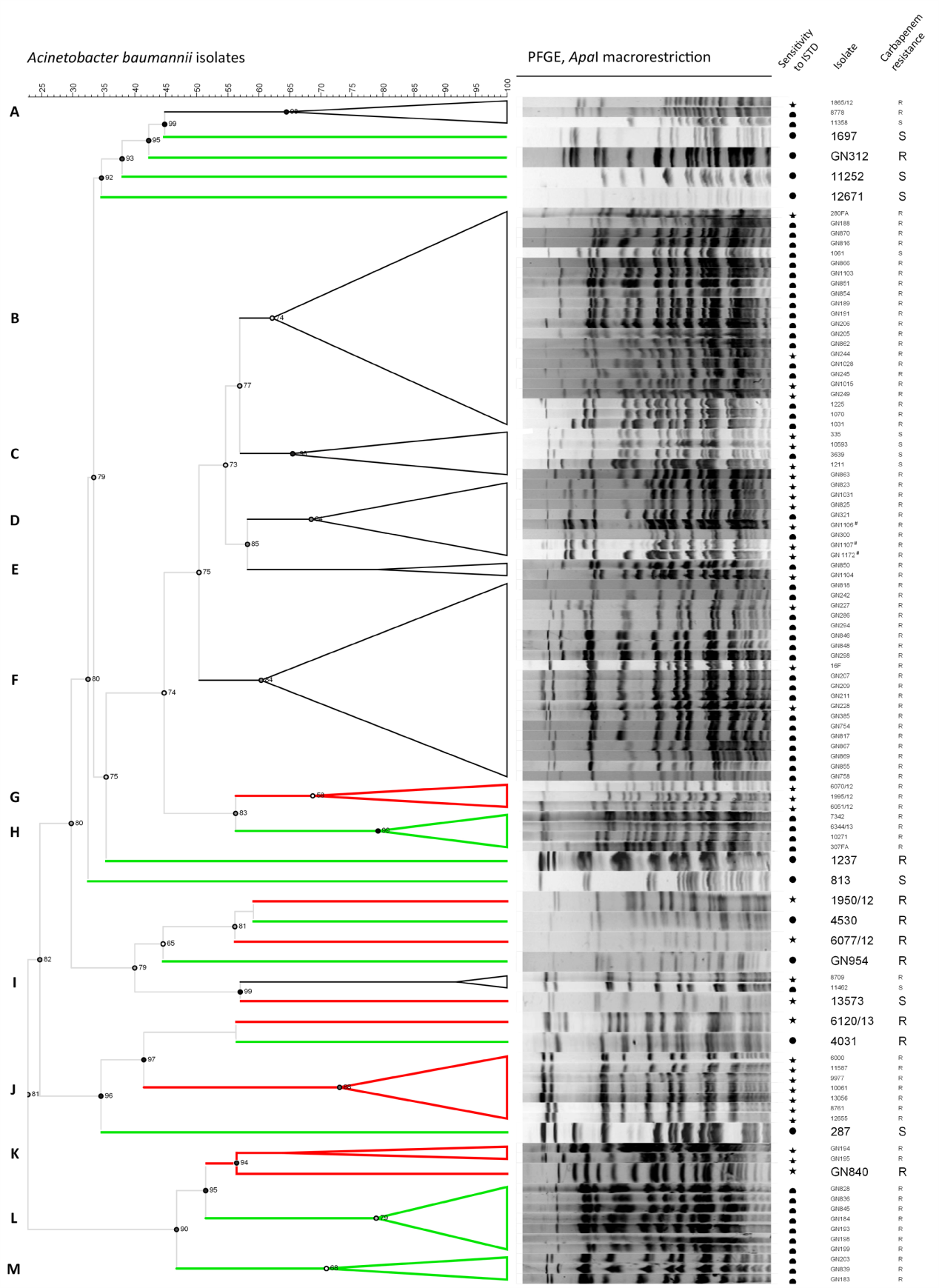
**

**Figure S1. PFGE dendrogram generated by BioNumerics 7.6 software, showing the diversity of fingerprints for 103 *A. baumannii* isolates**. On the left: Red color represents isolates sensitive to phage ISTD, green represents resistant ones. Black labeled clusters are consisted of both sensitive and resistant isolates. On the right, sensitivity to phage ISTD (star - sensitive, circle - resistant), isolates names, and sensitivities to carbapenems are listed. Colistin-resistant strains are labeled with ^#^


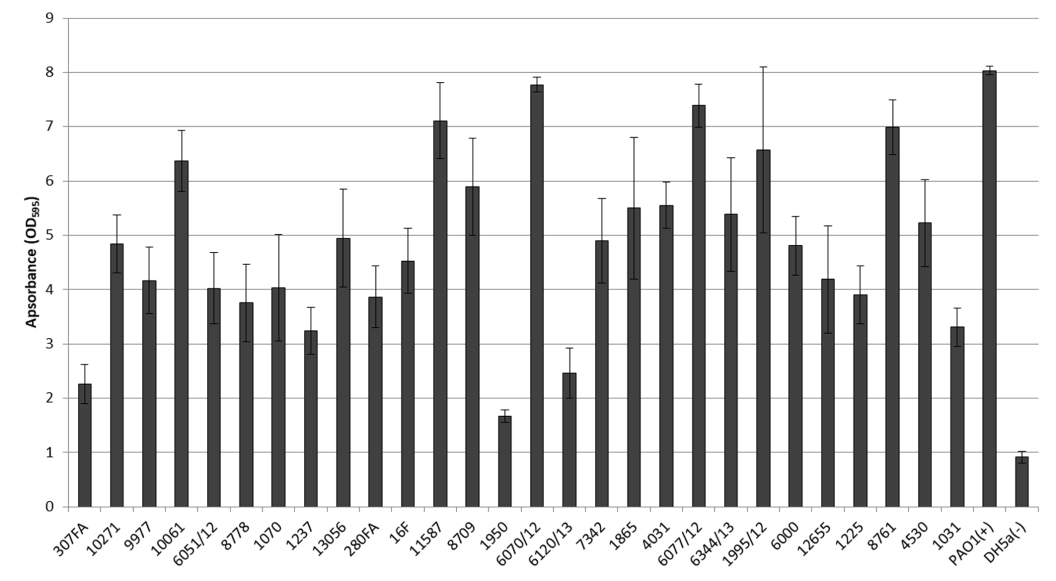


**Figure S2. Biofilm production of 28 carbapenem-resistant *A. baumannii* from laboratory collection.** Biofilm production was measured using the crystal violet assay. Data are mean values ± S.D. of decuplicate samples. *Pseudomonas aeruginosa* PAO1 and *E. coli* DH5α strains were used as positive and negative control.

**1.2** **Supplementary tables**

**Table S1.** **List of isolates used in the study**. Resistance to carbapenems, phage ISTD and phage NOVI is designated for each isolate. Colistin-resistant isolates are labeled with ^#^. PFGE typing results are given for all isolates, while European clonal complex is presented for the strains analyzed.
